# COX7A1 suppresses the viability of human non‐small cell lung cancer cells via regulating autophagy

**DOI:** 10.1002/cam4.2659

**Published:** 2019-10-30

**Authors:** Lei Zhao, Xin Chen, Yetong Feng, Guangsuo Wang, Imran Nawaz, Lifu Hu, Pengfei Liu

**Affiliations:** ^1^ Department of Anesthesiology The 2nd Clinical Medical College (Shenzhen People's Hospital) of Jinan University The 1st Affiliated Hospitals of Southern University of Science and Technology Shenzhen China; ^2^ Integrated Chinese and Western Medicine Postdoctoral Research Station Jinan University Guangzhou China; ^3^ Department of Laboratory Medicine The 2nd Clinical Medicine College (Shenzhen People's Hospital) of Jinan University The 1st Affiliated Hospitals of Southern University of Science and Technology Shenzhen China; ^4^ Department of Medicine College of Medicine University of Arizona Tucson AZ USA; ^5^ Department of Thoracic Surgery The 2nd Clinical Medicine College (Shenzhen People's Hospital) of Jinan University The 1st Affiliated Hospitals of Southern University of Science and Technology Shenzhen China; ^6^ Department of Microbiology, Tumor and Cell Biology Karolinska Institutet Stockholm Sweden

**Keywords:** autophagy, cell viability, COX7A1, non‐small cell lung cancer, NOX2, PGC‐1α

## Abstract

COX7A1 is a subunit of cytochrome c oxidase, and plays an important role in the super‐assembly that integrates peripherally into multi‐unit heteromeric complexes in the mitochondrial respiratory chain. In recent years, some researchers have identified that COX7A1 is implicated in human cancer cell metabolism and therapy. In this study, we mainly explored the effect of COX7A1 on the cell viability of lung cancer cells. COX7A1 overexpression was induced by vector transfection in NCI‐H838 cells. Cell proliferation, colony formation and cell apoptosis were evaluated in different groups. In addition, autophagy was analyzed by detecting the expression level of p62 and LC3, as well as the tandem mRFP‐GFP‐LC3 reporter assay respectively. Our results indicated that the overexpression of COX7A1 suppressed cell proliferation and colony formation ability, and promoted cell apoptosis in human non‐small cell lung cancer cells. Besides, the overexpression of COX7A1 blocked autophagic flux and resulted in the accumulation of autophagosome via downregulation of PGC‐1α and upregulation of NOX2. Further analysis showed that the effect of COX7A1 overexpression on cell viability was partly dependent of the inhibition of autophagy. Herein, we identified that COX7A1 holds a key position in regulating the development and progression of lung cancer by affecting autophagy. Although the crosstalk among COX7A1, PGC‐1α and NOX2 needs further investigation, our study provides a novel insight into the therapeutic action of COX7A1 against human non‐small cell lung cancer.

## Graphical abstract

COX7A1 could inhibit cell proliferation and colony formation ability, as well as promote cancer cell apoptosis. COX7A1 overexpression blocked the autophagic flux via downregulation of PGC‐1α and upregulation of NOX2.

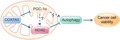

## 1 INTRODUCTION

Cytochrome c oxidase (COX) is a complex in the mitochondrial respiratory chain. To catalyze the reduction of molecular oxygen to water, the cytochrome c is used as a substrate by COX. This is the terminal and rate‐limiting step of mitochondrial respiration, which contributes to the energy stored in the electrochemical gradient.^1^, ^2^, ^3^ COX consists of 13 individual subunits, and one of these is subunit 7a (COX7A). COX7A also contains two different isoforms encoded by separate nuclear genes: COX7A1 and COX7A2. COX7A1 is located on chromosome 19q13.12, and it is most abundantly expressed in skeletal and heart muscles. In addition, this protein plays an important role in the super‐assembly that integrates peripherally into multi‐unit heteromeric complexes in the mitochondrial respiratory chain.^4^, ^5^ The generation of mitochondrial energy is essential for the function of cardiac tissue, and some researchers have found that the mice lacking both homozygous and heterozygous heart‐type COX7A1 develop dilated cardiomyopathy at 6 weeks of age,^6^ indicating the key position of COX7A1 in energy generation and metabolism.

In recent years, scientists have noticed that mitochondrial dysfunction is implicated in multiple diseases, especially for cancer.^7^, ^8^ Among all kinds of different cancers, lung cancer is considered as the most common type all over the world. Therefore, it could be possible that different COX subunits are implicated in the metabolism of cancer cells although the detailed molecular and regulatory mechanisms remain unclear. In 2017, a novel function of COX7A1 was first identified in human lung adenocarcinoma cells. Mishra et al analyzed the expression of different stages of lung adenocarcinoma using Gene Expression Omnibus datasets and found that the expression of COX7A1 gene was highly down‐regulated in patients with lung cancer. Further results showed that the overexpression of COX7A1 inhibited cell proliferation ability and increased cell apoptosis. Therefore, this research indicated that the low expression of COX7A1 gene may be essential to maintain the cell viability of lung cancer cells.^9^

Autophagy is a type of bulk degradation process, as well as a mechanism of intracellular protein and organelle recycling. In this process, the cell engulfs proteins, protein aggregates, organelles or lipids go into a double‐membrane vesicle, autophagosome, which will further fuses with the lysosome to form the autolysosome finally.^10^ For cancer cells, autophagy is indispensable because of the high self‐renewal capacity, and the block of autophagic flux affects the cell viability of cancer cells and results in the increase of reactive oxygen species (ROS) production and metabolic abnormalities. However, the activation of autophagy showed some tumor suppressive properties, since autophagy can reduce oxidative stress, suppress oncogenic signaling, and limits genome instability, blocking the initiation of cancer.^11^, ^12^, ^13^, ^14^ Therefore, the role of autophagy in cancer development and therapy is still in debate.

NOX2 is a superoxide generating enzyme, which forms ROS.^15^ In addition, the relationship between NOX2 and autophagy has also been investigated by researchers. For example, scientists found that the activation of NOX2 could block autophagic flux by impairing lysosomal enzyme activity, and the inhibition of NOX2 could suppress the overproduction of superoxide, and restore the lysosome acidification as well as its enzyme activity, thereby reducing the accumulation of autophagosomes.^16^ Moreover, some evidences also demonstrated that the enhanced reactive oxygen species production by NOX2 could impair autophagy in muscles, while the decrease of CYBB/NOX2‐mediated oxidative stress could enhance autophagy induction.^17^, ^18^ Therefore, NOX2 holds a key position in the regulation of autophagy.

In this study, we mainly explored the effect of COX7A1 on cell viability and autophagy in human non‐small cell lung cancer cells. Our results indicated that COX7A1 suppressed cell proliferation capacity and colony formation ability, and promoted cell apoptosis. In addition, the overexpression of COX7A1 blocked autophagic flux via downregulation of PGC‐1α and upregulation of NOX2, and further analysis showed that the effect of COX7A1 on cell viability was partly dependent on the inhibition of autophagy. Therefore, our study identified that COX7A1 plays a crucial role in the treatment of human non‐small cell lung cancer.

## 2 MATERIALS AND METHODS

### 2.1 Cell culture

Human non‐small cell lung cancer cell lines, NCI‐H838 and NCI‐H1703, were purchased from the American Type Culture Collection (ATCC). The cells were cultured with RPMI‐1640 (Gibco) supplemented with 10% Fetal Bovine Serum (Gibco), 100 U/mL penicillin and 0.1 g/mL streptomycin (Sigma) in a humidified 37°C incubator with 5% CO_2_. The culture medium was replaced every other day, and the H838 cells were passaged (dilution, 1:4) every 5 or 6 days.

### 2.2 Overexpression of COX7A1 in cancer cells

The coding sequence of human COX7A1 was amplified using PCR (Phusion® High‐Fidelity DNA Polymerase, New England BioLabs) and subsequently cloned into a pCI vector (Promega Corporation). To overexpress COX7A1 in H838 cells, the cells were transfected with the pCI‐COX7A1 vector (final concentration: 2 μg/mL) using Lipofectamine 3000 (Invitrogen) according to the manufacturer's protocol. The control group for COX7A1 overexpression was performed with the transfection of empty vector. Following transfection for 24 hours, the H838 cells in different groups were harvested for subsequent experimentation. The overexpression efficiency was examined using western blot.

### 2.3 Cell proliferation assay

To evaluate cell proliferation ability, the proliferation index was measured in different groups using CCK‐8 method (Dojindo, Japan) as the references.^19^, ^20^, ^21^ Briefly, 20 μL of the CCK‐8 solution was added into different wells, which contained 200 μL of medium, and was further incubated at 37°C for 4 hours. The absorbances (Abs) at 450 nm were detected respectively (n = 3). The wells containing only RPMI‐1640 medium were used as the blank group. Herein, the proliferation index = Abs of the experimental group—Abs of the blank group, was used to evaluate cell proliferation ability.

### 2.4 Real‐time qRT‐PCR

Total mRNA in each group was extracted using TRIzol (Invitrogen) according to the manufacturer's instructions. Then, cDNA was synthesized using 2 μg of mRNA and a Transcriptor first‐strand cDNA synthesis kit (Promega). Real‐time qPCR was then performed as previously described.^19^, ^20^, ^21^ β‐actin was used for qPCR normalization, and all experiments were measured in triplicate. Primer sequences (5′‐3′) are as follows:

*p62*‐ Forward 5′‐GACTACGACTTGTGTAGCGTC‐3′.

*p62*‐ Reverse 5′‐AGTGTCCGTGTTTCACCTTCC‐3′.

*β‐actin*‐ Forward 5′‐CCCAGAGCAAGAGAGG‐3′.

*β‐actin*‐ Reverse 5′ ‐GTCCAGACGCAGGATG‐3′.

### 2.5 Western blot

In our study, radioimmunoprecipitation assay lysis buffer (RIPA, Thermo Fisher Scientific) was used to extract the total protein. The protein concentration in different groups was measured using a Pierce BCA Protein Assay Kit (Thermo Fisher Scientific). Herein, the western blot assay was performed as previously described.^22^, ^23^, ^24^ In brief, the protein sample (15 µg/lane) was separated using 10% Sodium Dodecyl Sulfate‐Polyacrylamide Gel Electrophoresis gel and then transferred to nitrocellulose membranes. After blocking with 5% bovine serum albumin (Sigma), the membrane was incubated with primary antibodies overnight at 4°C. In this study, the primary antibodies used were: anti‐COX7A1 (1:3000; Abcam, ab123591), anti‐Bax (1:3000; Abcam, ab53154), anti‐Caspase 3 (1:3000; Abcam, ab13847), anti‐PGC‐1α (1:3000; Abcam, ab54481), anti‐PGC‐1β (1:1000; Santa Cruz, sc‐373771), anti‐RIP140 (1:2000; Abcam, ab91476), anti‐p62 (1:3000; Abcam, ab56316), anti‐LC3 (1:3000; Sigma, L7543), anti‐NOX2 (1:3000; Abcam, ab31092) and anti‐GAPDH (1:3000; Santa Cruz, sc‐47724). The secondary antibodies used were: Anti‐mouse IgG (HRP‐conjugated; 1:5000; Sigma‐Aldrich, A‐9044) and anti‐rabbit IgG (HRP‐conjugated; 1:5000; Sigma‐Aldrich, A‐0545). Finally, the protein bands were visualized using an enhanced chemiluminescence kit (SuperSignal West Femto Maximum Sensitivity Substrate, Thermo Fisher Scientific) and ChemiDoc Imagers (Bio‐Rad Laboratories). The results were quantified using ImageJ 1.x software (National Institutes of Health).

### 2.6 Colony formation assay

In the colony formation assay, 500 cells were seeded into 12‐well plates. The cells were incubated for 7 days in the incubator at 37°C with 5% CO_2_. Then, the cells were fixed for 20 minutes at room temperature using 4% paraformaldehyde, and stained with crystal violet (5 mg/mL) for 20 minutes at room temperature. Finally, the cell colonies were imaged using an Epson Perfection V600 scanner (Seiko Epson Corporation).

### 2.7 Cell apoptosis assay

The expression levels of apoptotic genes (Bax and Caspase 3) in different groups were measured using western blot. In addition, cell apoptosis was further analyzed using Annexin V/PI Cell Apoptosis Assay kit (Thermo Fisher Scientific) and Tunel‐FITC Assay Kit (Abcam) according to the manufacturer's protocol. In brief, the cells in each group were dissociated into single cells using trypsin, and washed with PBS. Then, the cell samples were incubated with different staining solutions. Finally, cell samples were detected using a FACSCalibur flow cytometer and FlowJo 7.6.1 software.

### 2.8 Small interfering RNAs (siRNAs) transfection

To knockdown PGC‐1α and NOX2, siRNAs for different genes (siRNA for PGC‐1α: SI00101031; siRNA for NOX2: SI00008729) were purchased from QIAGEN. All of the siRNAs were transfected into cancer cells using Lipofectamine 3000 (Invitrogen) according to the manufacturer's protocol. The cells in the negative control group were transfected with a scrambled sequence of the siRNA. The knockdown effect of siRNA was evaluated using western blot.

### 2.9 Live cell immunofluorescence microscopy

In our study, ptf‐LC3 vector (mRFP‐GFP‐LC3 reporter construct) was purchased from Addgene, USA. H838 cells were grown in 35 mm glass‐bottom dishes. On reaching 50% confluence, the cells were transfected with different vectors using Lipofectamine 3000 for 24 hours. Then, the cells in different groups were imaged in phenol red‐free medium using a Zeiss Observer Fluorescence Microscope.

### 2.10 Statistical analysis

The results are presented as mean ± SD. All statistical analysis was performed using SPSS 17.0. Herein, unpaired Student's *t* tests were applied to compare the means of two groups, and one‐way ANOVA with Bonferroni's correction was used to compare the means of three or more groups. One‐tailed test was used in the Student's *t* test. *P* < .05 was considered statistically significant.

## 3 RESULTS

### 3.1 COX7A1 overexpression inhibits cell viability and promotes cell apoptosis in human lung cancer cells

**Figure 1:**
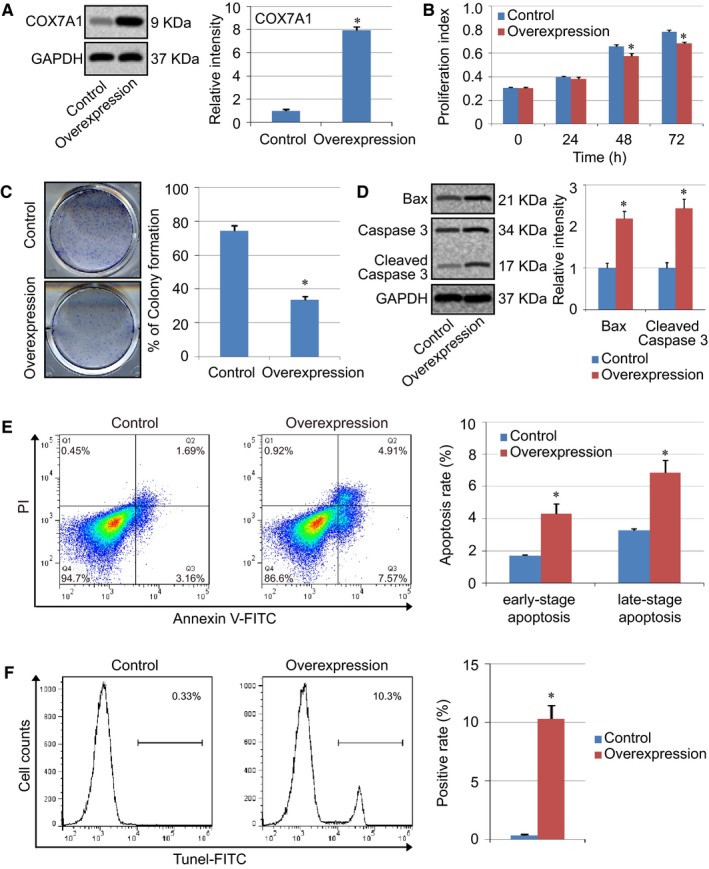
The overexpression of COX7A1 suppressed cell viability and promoted cell apoptosis in human H838 cells. A, Western blot detection of COX7A1 overexpression induced by vector transfection. B, Evaluation of cell proliferation in the Control and COX7A1 overexpression group. C, Colony formation ability assay. D, Detection of apoptosis genes (Bax and Caspase 3) using western blot. E and F, Cell apoptosis assay using Annexin V‐FITC/PI staining (E) and Tunel staining (F). H838 cells were transfected with pCI‐COX7A1 to induce the overexpression of COX7A1, and an empty vector was used in the control group. The cells were harvested for detection after being transfected for 24 hours. Results are expressed as mean ± SD. A *t* test was used to compare the different groups, and *P* < .05 was considered statistically significant. *P < .05 compared with the Control group

In this study, human H838 lung cancer cells were tranfected with pCI‐COX7A1 to induce COX7A1 overexpression, and western blot results showed that the expression level of COX7A1 in the Overexpression group was much higher (about 8‐fold) than the Control group (Figure 1A). Cell proliferation ability was measured using CCK‐8 at different time points, and the results indicated that the proliferation index of COX7A1 in the Overexpression group was lower than that of the Control group after 48 hours (Figure 1B). In addition, the cell viability of different groups was further evaluated using the colony formation assay. The results showed that the number of colonies in Control group was much higher than the COX7A1 Overexpression group, indicating the inhibitory capability of COX7A1 on the cell viability of human non‐small cell lung cancer cells (Figure 1C).

Cell apoptosis was evaluated in our study. The western blot results showed that the expression of both Bax and cleaved Caspase 3 was increased with COX7A1 overexpression (Figure 1D). In addition, the effect of COX7A1 on cell apoptosis was also measured using Annexin V/PI staining and Tunel staining. The percentage of both early‐stage apoptotic cells (Annexin V‐positive and PI‐negative) and late‐stage apoptotic cells (Annexin V‐positive and PI‐positive) was analyzed by flow cytometry, and the results indicated that COX7A1 overexpression increased the percentage of both early‐stage and late‐stage apoptotic cells, which was consistent with the western blot results and Tunel staining results about cell apoptosis (Figure 1E,F).

### 3.2 COX7A1 induces the block of autophagy via downregulation of PGC‐1α

**Figure 2:**
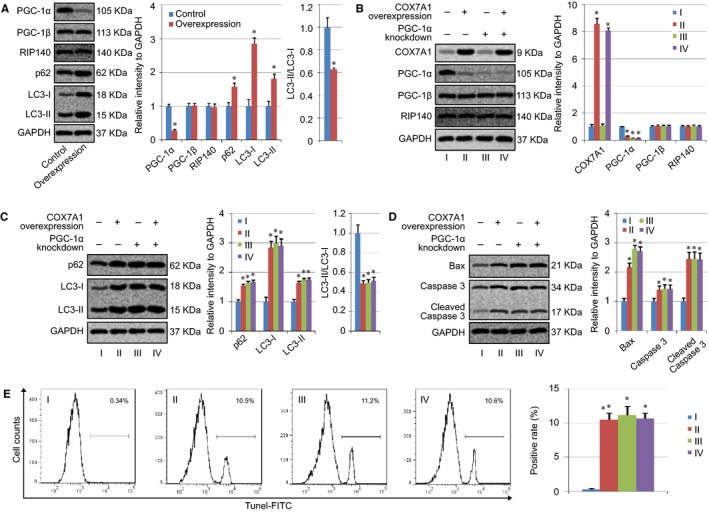
COX7A1 induced the blocking of autophagy via downregulation of PGC‐1α. A, Effect of COX7A1 on PGC‐1, RIP140, and autophagy‐related proteins. B, Evaluation of PGC‐1α knockdown using western blot. C, Effect of PGC‐1α knockdown on the level of autophagy‐related proteins. D, Detection of apoptosis genes (Bax and Caspase 3) expression. E, Cell apoptosis assay using Tunel staining. Results are expressed as mean ± SD. A *t* test was used to compare the different groups, and *P* < .05 was considered statistically significant. *P < .05 compared with the Control group (group I)

A study has indicated the effect of COX7A1 on PGC‐1 in skeletal muscle cells, and the COX7A1 knockout increased the expression level of PGC‐1β.^25^ Therefore, both PGC‐1α and PGC‐1β were detected in our research together with RIP140, the inhibitor of PGC‐1. We found that the expression of PGC‐1β and RIP140 was not affected by COX7A1 overexpression, while the level of PGC‐1α was decreased (Figure 2A). As the key function of PGC‐1α in autophagy has been identified by some researchers,^26^ the autophagy‐related proteins, p62 and LC3 were further detected in our study using western blot. The results showed that the protein levels of p62, LC3‐I and LC3‐II were increased in COX7A1 Overexpression groups (Figure 2A), but not the mRNA level of p62 (Figure S1). However, the ratio of LC3‐II/LC3‐I in the COX7A1 Overexpression groups was much lower than that of the Control group, indicating that COX7A1 might inhibit autophagy in human lung cancer cells (Figure 2A).

To confirm the role of PGC‐1α in the COX7A1‐regulated autophagy, PGC‐1α knockdown was induced by PGC‐1α siRNA transfection, and western blot detection showed that the PGC‐1α level was much lower in the different groups after transfection with siRNA. In addition, the level of PGC‐1β or RIP140 did not show any change after transfection (Figure 2B). p62 and LC3 were also measured herein, and we found that the knockdown of PGC‐1α showed a similar effect as that of COX7A1 Overexpression, and increased the protein level of both p62 and LC3. However, no difference in p62 and LC3 expression could be found between the Control group and COX7A1 Overexpression group in PGC‐1α‐knockdown lung cancer cells (Figure 2C). In addition, cell apoptosis was evaluated in different groups as well. We found the expression of both Bax and cleaved Caspase 3, and Tunel positive rate were up‐regulated in H838 cells transfected with PGC‐1α siRNA (Group I v.s. Group III), and the level of the cell apoptosis didn't show obvious difference between the Control group and COX7A1 Overexpression group in PGC‐1α‐knockdown lung cancer cells (Group III vs. Group IV), which was consistent with the autophagy evaluation (Figure 2D,E).

### 3.3 COX7A1 overexpression results in the accumulation of autophagosomes

**Figure 3:**
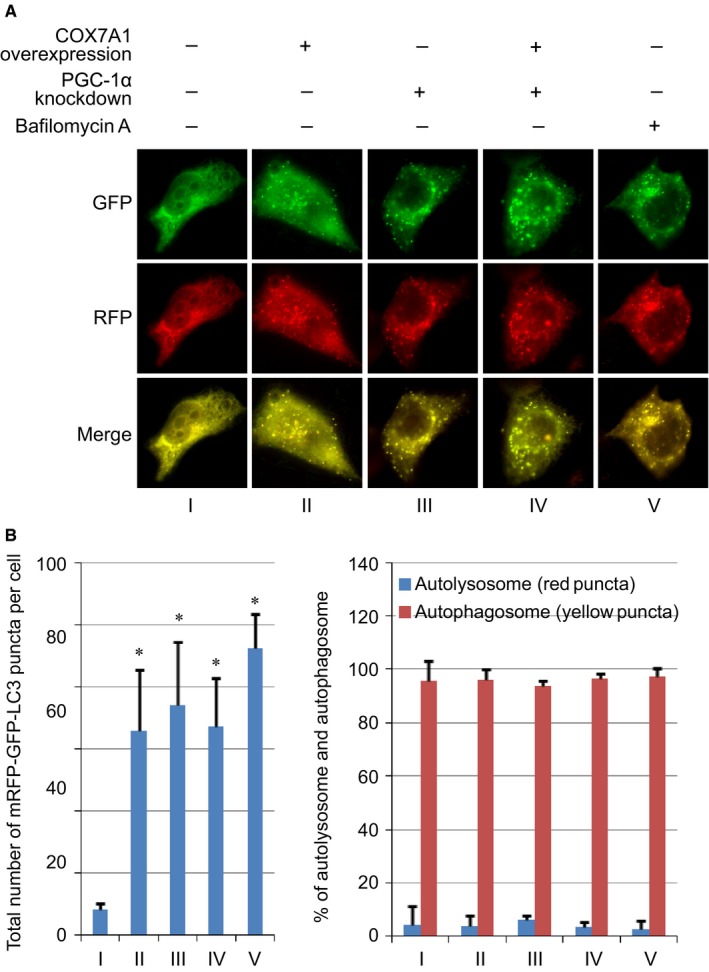
Evaluation of autophagic flux using tandem mRFP‐GFP‐LC3 reporter. A, The fluorescence image of H838 cells after different transfection. Scale bar = 10 μm. B, Bar graphs represent the total number of mRFP‐GFP‐LC3 positive puncta and % of autolysosomes (red puncta) and autophagosome (yellow puncta) per cell. H838 cells were transfected with PGC‐1α siRNA first. After 24 hours, the cell were further transfected with pCI‐COX7A1 or ptf‐LC3 for another 24 hours. Then the cell samples in each group were applied for analysis. Results are expressed as mean ± SD. A *t* test was used to compare the different groups, and *P* < .05 was considered statistically significant. *P < .05 compared with the Control group (group I)

Besides, the effects of COX7A1 and PGC‐1α on autophagy were further detected using the tandem mRFP‐GFP‐LC3 reporter construct, which allowed us to analyze autophagy completion. Herein, the yellow puncta indicate autophagosomes, and the red puncta indicate autolysosomes due to the quenching of GFP in the acidic autolysosomal environment. The results indicated that the total number of mRFP‐GFP‐LC3 puncta (yellow puncta with few red puncta) was increased in the COX7A1 Overexpression group (group II) compared with the Control group (group I), and PGC‐1α knockdown abolished the difference between the COX7A1 Overexpression group and Control group. However, the percentage of autolysosome and autophagosome did not show significant any difference among each group (Figure 3A,B).

We noticed that the ratio of autolysosomes was low in normal H838 cells (group I). It could be possible that the blockage of autophagy in H838 cells is hard to further downregulate the ration of autolysosomes. To confirm this hypothesis, we also performed a positive control group treated with Bafilomycin A, which is a autophagy inhibitor and has been demonstrated to block the autophagic flux. In this group, the amount of autophagosomes was increased. However, the ratio of autolysosome also did not show significant difference compared with the control group without any treatment, which was similar to the results of the COX7A1 overexpression group (Figure 3A,B). Therefore, the overexpression of COX7A1 may block the autophagic flux and result in the accumulation of autophagosome, and this effect could be dependent on the downregulation of PGC‐1α.

The upregulation of NOX2 is essential for the influence of COX7A1 on autophagy.

**Figure 4:**
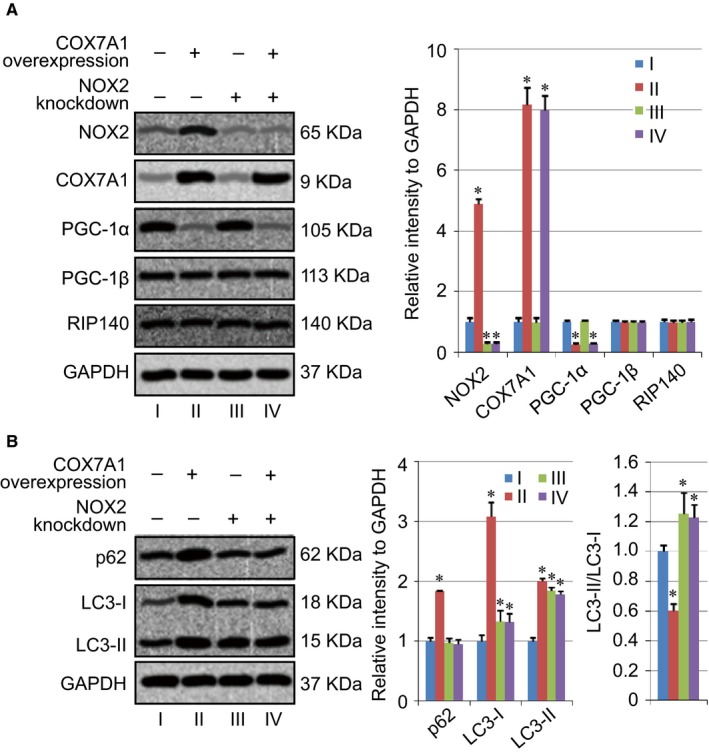
NOX2 knockdown abolishes the blockage of autophagy induced by COX7A1 overexpression. A, Evaluation of NOX2 knockdown using western blot. B, Effect of NOX2 knockdown on the level of autophagy‐related proteins. H838 cells were transfected with NOX2 siRNA first. After 24 hours, the cells were further transfected with pCI‐COX7A1 for another 24 hours to induce COX7A1 overexpression. Then the cell samples in each group were applied for analysis. Results are expressed as mean ± SD. A *t* test was used to compare the different groups, and *P* < .05 was considered statistically significant. *P < .05 compared with the Control group (group I)

NADPH oxidase isoform 2 (NOX2) is a type of superoxide generating enzyme complex, which forms ROS and is expressed in myeloid cells, and some cancer cells.^15^ A study has indicated that the activation of NOX2 blocked autophagic flux by impairing lysosomal enzyme activity.^16^ In our study, the role of NOX2 in COX7A1‐regulated autophagy was also analyzed, and we found that the level of NOX2 was increased in the COX7A1 Overexpression group (group II) compared with the Control group (group I). Therefore, knockdown of NOX2 was induced in both Control group and COX7A1 Overexpression group, and NOX2 knockdown did not affect the downregulation of PGC‐1α which was induced by COX7A1 overexpression, and the level of PGC‐1β or RIP140 did not change after NOX2 knockdown as well (Figure 4A). Moreover, the expression level of autophagy‐related proteins, p62 and LC3 was also detected herein. We found that the NOX2 knockdown increased the level of LC3‐I and LC3‐II, as well as the ratio of LC3‐II/LC3‐I. However, the expression of p62 did not show any change after NOX2 knockdown. In addition, the results also indicated that the overexpression of COX7A1 did not affect autophagy level in NOX2‐knockdown cells, indicating the key role of NOX2 in COX7A1‐induced autophagy inhibition (Figure 4B).

### 3.4 The influence of COX7A1 on cell viability partly depends on the regulation of NOX2

**Figure 5:**
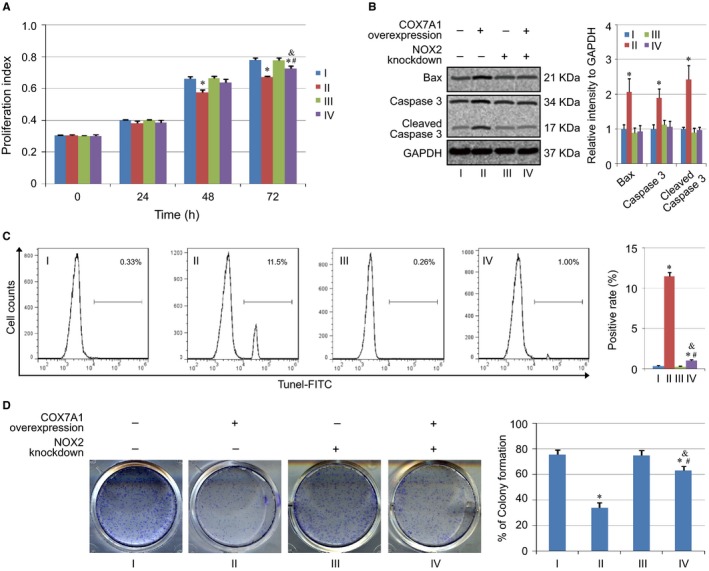
The influence of COX7A1 on cell viability depends on the regulation of NOX2 partly. A, Evaluation of cell proliferation in different groups. B, Detection of apoptosis genes (Bax and Caspase 3) expression in each group. C, Cell apoptosis assay using Tunel staining. D, Colony formation ability assay. Results are expressed as mean ± SD. A *t* test was used to compare the different groups, and *P* < .05 was considered statistically significant. *P < .05 compared with Group I. #: *P* < .05 compared with Group II. &: *P* < .05 compared with Group III

The influence of COX7A1 on cell viability was further evaluated in NOX2‐knockdown cells. The results indicated that the inhibition of cell proliferation induced by COX7A1 overexpression could be rescued by NOX2 knockdown in some degree (Figure 5A). Cell apoptosis was analyzed using western blot and Tunel staining, and we noticed that NOX2 knockdown did not alter the expression level of Bax and Caspase 3, as well as Tunel positive rate (Group I vs Group III). However, the overexpression of COX7A1 could not induce the upregulation of Bax and Caspase 3, and could only induce a tiny increase in Tunel positive rate in NOX2‐knockdown cells (Group III vs. Group IV) (Figure 5B,C). At last, the cell viability of each group was further evaluated using colony formation assay. Our results indicated that the colony formation ability was not affected by NOX2 knockdown. Similar to proliferation results, the inhibition of COX7A1 overexpression on colony formation ability was rescued in some degree by NOX2 knockdown, indicating that the influence of COX7A1 on cell viability depends on the regulation of autophagy partly (Figure 5D).

## 4 DISCUSSION

In recent years, scientists have demonstrated that the regulation of energy generation and cell cycle progression in cancer cells are different from normal cells, and the energy homeostasis also varies in different types of cancers.^27^, ^28^ Most cancer cells can reserve the capacity to operate oxidative phosphorylation in normoxic conditions, and thrive on glycolysis, which is defined as the classical concept of the “Warburg effect”.^29^, ^30^, ^31^ Furthermore, a study also indicated that the mitochondrial oxidative metabolism holds a promising potential in the metabolic therapy against tumor metastasis.^32^ The role of COX subunits has also been investigated in several types of cancers.^9^, ^33^, ^34^ For example, Mishra et al compared the expression of different COX subunit genes in human lung adenocarcinoma tissues with that of normal lung tissues using available microarray database, and the results showed that the expression of COX7A1 was much lower in the cancer tissues than in normal lung tissues, suggesting the possibility that COX7A1 inhibited the development of lung cancer.^9^ In our study, our results indicated that the overexpression of COX7A1 could inhibit cell proliferation and increase cell apoptosis in human non‐small cell lung cancer cells. Further analysis indicated that the effect of COX7A1 on lung cancer cell viability was partly dependent on the regulation of autophagic flux.

Autophagy is considered as a survival‐promoting pathway. In this process, the intracellular proteins and organelles can be captured, degraded and recycled in lysosomes, which release the toxic buildup of cellular waste products, and provide substrates to support the metabolism in starvation. During the process of cancer development, autophagy is up‐regulated to make cancer cells survive the microenvironmental stress. In addition, the upregulation of autophagy also promotes the growth and aggressiveness of cancer cells.^35^ The possible mechanism by which autophagy promotes the development of cancer could include inhibiting the function of p53 cancer suppressor protein and maintaining the metabolic function of mitochondria.^36^, ^37^ Therefore, improving cancer therapy via inhibition of autophagy has attracted great interest in recent years. However, scientists also notice that the defective autophagy in normal cells is associated with genomic instability as well as tumorigenesis.^38^ For example, mice with deficiency of Atg5 and Atg7 easily develop liver cancer because of oxidative stress and mitochondrial damage.^39^ Therefore, autophagy plays a significant role in the process of cancer development and progression, with both cancer‐suppressive and cancer‐promoting function. In our study, the critical involvement of autophagy in COX7A1‐mediated apoptosis and inhibition of proliferation may further be confirmed by stimulating autophagy. However, nearly all autophagy inducers such as Rapamycin,^40^ Simvastatin 41 and Amiodarone possess anticancer effect and inhibit cancer cell viability.^42^ It is hard to find any functional autophagy inducer which does not affect cancer cell viability. Therefore, a more specific autophagy inducer without anticancer effect is necessary for cancer research.

Some researchers have indicated the effect of COX7A1 on PGC‐1 in skeletal muscle cells, and their results showed that the COX7A1 knockout increased the expression level of PGC‐1β.^25^ Therefore, both PGC‐1α and PGC‐1β were detected in our research together with RIP140, the inhibitor of PGC‐1. Different from the report, our results indicated that the expression of PGC‐1β and RIP140 was not affected by COX7A1 overexpression, while the level of PGC‐1α was decreased (Figure 2A). The key function of PGC‐1α in autophagy has been identified by some researchers. They compared PGC‐1α knockout mice with wild‐type mice, and found that the deletion of PGC‐1α led to a 25% decline in running performance, and a 40% decrease in mitochondrial content. In addition, exercise could enhance the targeting of mitochondria for mitophagy, and increase the autophagy and mitophagy flux in wild‐type mice, but not in PGC‐1α knockout mice, indicating the key role of mitochondrial turnover and PGC‐1α in autophagy regulation.^26^

NOX2 is a superoxide generating enzyme, which forms ROS.^15^ In addition, the effect of NOX2 on autophagy also has been investigated by researchers.^16^ Scientists found that the activation of NOX2 could block autophagic flux by impairing lysosomal enzyme activity, and the inhibition of NOX2 could suppress the overproduction of superoxide and restore the lysosome acidification as well as its enzyme activity, further reducing the accumulation of autophagosome.^16^ Our results indicated that the influence of COX7A1 on autophagy is mainly based on the regulation of NOX2. COX7A1 overexpression did not show any effect on autophagy level in NOX2 knockdown cells. Moreover, we also found that the knockdown of NOX2 increased the ratio of LC3‐II/LC3‐I, indicating that the downregulation of NOX2 might promote autophagy in human lung cancer cells.

However, the detailed mechanism of the effect of COX7A1 on PGC‐1 and NOX2 is still unclear for us. Similar to COX7A1, NOX2 also holds an important role in the process of mitochondrial energy metabolism.^43^, ^44^, ^45^ A study has indicated that COX7A1 can regulate the level of PGC‐1 in skeletal muscle cells,^25^ and PGC‐1 can promote eNOS expression and activity, which holds a key potential in ROS regulation.^46^ The ROS reduces the eNOS activity and increases nitric oxide consumption. Moreover, an important source of ROS is a family of NADPH oxidases, such as NOX2.^47^ Therefore, the crosstalk among COX7A1, PGC‐1 and NOX2 could be through cellular ROS regulation and negative feedback in mitochondria. The ROS level was also evaluated via FACS in our study. However, no difference could be found between control group and COX7A1 overexpression group (Figure S2), indicating the potential position of negative feedback in the mitochondria.

**Figure 6:**
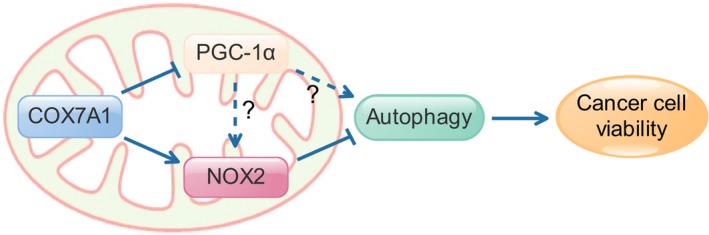
Proposed model for the function of COX7A1 to suppressing the viability of human non‐small cell lung cancer cells via regulating autophagy. Autophagy could be inhibited by NOX2, and activated by PGC‐1α. Herein, the effect of COX7A1 on NOX2 and PGC‐1 is different. COX7A1 overexpression leads to the downregulation of PGC‐1α and upregulation of NOX2, which further results in the inhibition of autophagy totally

In addition, our results indicated that the overexpression of COX7A1 decreased the expression of PGC‐1 and increased the expression of NOX2. However, the relationship between PGC and NOX2 is unclear. We noticed that the knockdown of NOX2 did not affect the expression level of PGC‐1, while the downregulation of PGC‐1 expression induced by COX7A1 overexpression did not show any effect on autophagy in NOX2 knockdown cells. These results suggested that the effect of PGC‐1 on autophagy might depend on the normal expression of NOX2, and NOX2 could be the protein downstream of PGC‐1 in COX7A1‐induced autophagy regulation (Figure 6). Needless to say, this hypothesis still needs more evidence for confirmation.

In this study, our results indicated that the inhibition of COX7A1 overexpression on cell viability was rescued in some degree by NOX2 knockdown, indicating that the influence of COX7A1 depends on the regulation of autophagy partly. As COX7A1 is a subunit in mitochondrial respiratory chain, it could be possible that COX7A1 regulates cell viability by affecting the mitochondrial respiratory chain and energy generation directly. For example, Mishra et al found that the overexpression of COX7A1 in human lung cancer cells (A549) could lead to the inhibition of cell proliferation and the increase in cell apoptosis. To evaluate whether autophagy could be involved in COX7A1‐mediated cell death, they further detected the expression level of BECN1, a gene which holds a key role in autophagy. However, the results showed that no change in the expression level of BECN1 could be observed after COX7A1 overexpression, suggesting that the cell death induced by COX7A1 overexpression might not be due to autophagy regulation in A549 cells.^9^ In our research, we mainly analyzed autophagy by detecting the expression level of p62 and LC3, as well as the tandem mRFP‐GFP‐LC3 reporter assay in another human non‐small cell lung cancer cell line, NCI‐H838. Our results showed that the overexpression of COX7A1 may block the autophagic flux and result in the accumulation of autophagosome. Besides, we further explored the function of COX7A1 in another non‐small cell lung cancer cell line, NCI‐H1703, and the results were similar to the previous results from H838 cells, indicating the negative regulation effect of COX7A1 on cancer cell viability and autophagy (Figures S3 and S4). We noticed that both H838 and H1703 showed a low expression of COX7A1. Another group also indicated the low expression of COX7A1 in A549 lung cancer cells, as well as in lung cancer tissue.^9^ It could be possible that most non‐small cell lung cancer cell lines hold a low expression of COX7A1, and the function of COX7A1 could be different in the NSCLC cell line with high expression of COX7A1 compared with the NSCLC cell line with low expression of COX7A1. Anyway, more cells lines are needed to confirm the accurate effect of COX7A1 on autophagy. Especially, the in vivo study is more reliable than the in vitro data. However, only several groups have explored the function of COX7A1 in different disease or metabolism models, and no COX7A1 inducers or inhibitors have been developed so far, which are necessary to regulate COX7A1 level in vivo. Therefore, maybe scientists should make more effects on COX7A1 inducer or inhibitor screen, which is significant for the preclinical study of COX7A1 in different disease models as well as dose‐dependent effect assay. A better understanding of the detailed function and the related mechanism of COX7A1 in different types of cell and animal models should promote the development of novel and effective methods for lung cancer therapy.

## 5 CONCLUSION

In conclusion, our study mainly addressed the link between COX7A1 and hallmarks of human non‐small cell lung cancer cells. The results indicated that the overexpression of COX7A1 could inhibit cell proliferation and colony formation ability, as well as promote cancer cell apoptosis. In addition, COX7A1 overexpression blocked the autophagic flux via downregulation of PGC‐1α and upregulation of NOX2, and the influence of COX7A1 on cell viability depends on the regulation of autophagy partly. Although the crosstalk among COX7A1, PGC‐1α and NOX2 needs our further investigation, our study provides a novel insight into the therapeutic action of COX7A1 against human human non‐small cell lung cancer.

## CONFLICTS OF INTEREST

The authors declare that there are no conflicts of interest.
