# Supplementary figures and images for: COX7A1 suppresses the viability of human non‐small cell lung cancer cells via regulating autophagy
Source: Cancer Med. 2019 Oct 30;8(18):7762–73. doi: 10.1002/cam4.2659 (PMC6912042; doi:10.1002/cam4.2659)

# Figure S1

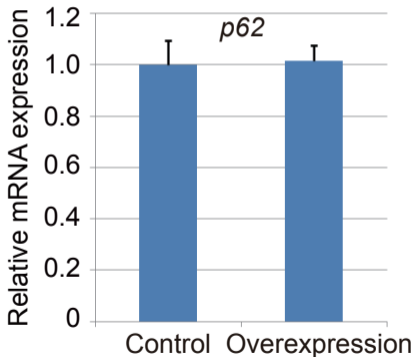

Supplement: Supplementary file 1 [file CAM4-8-7762-s001.pdf]

# Figure S2

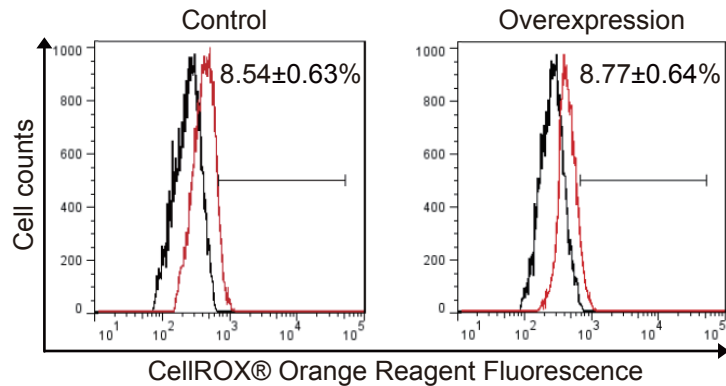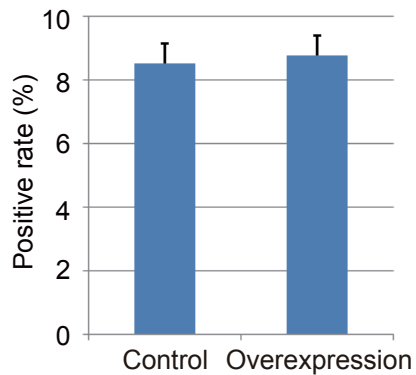

Supplement: Supplementary file 2 [file CAM4-8-7762-s002.pdf]

# Figure S3

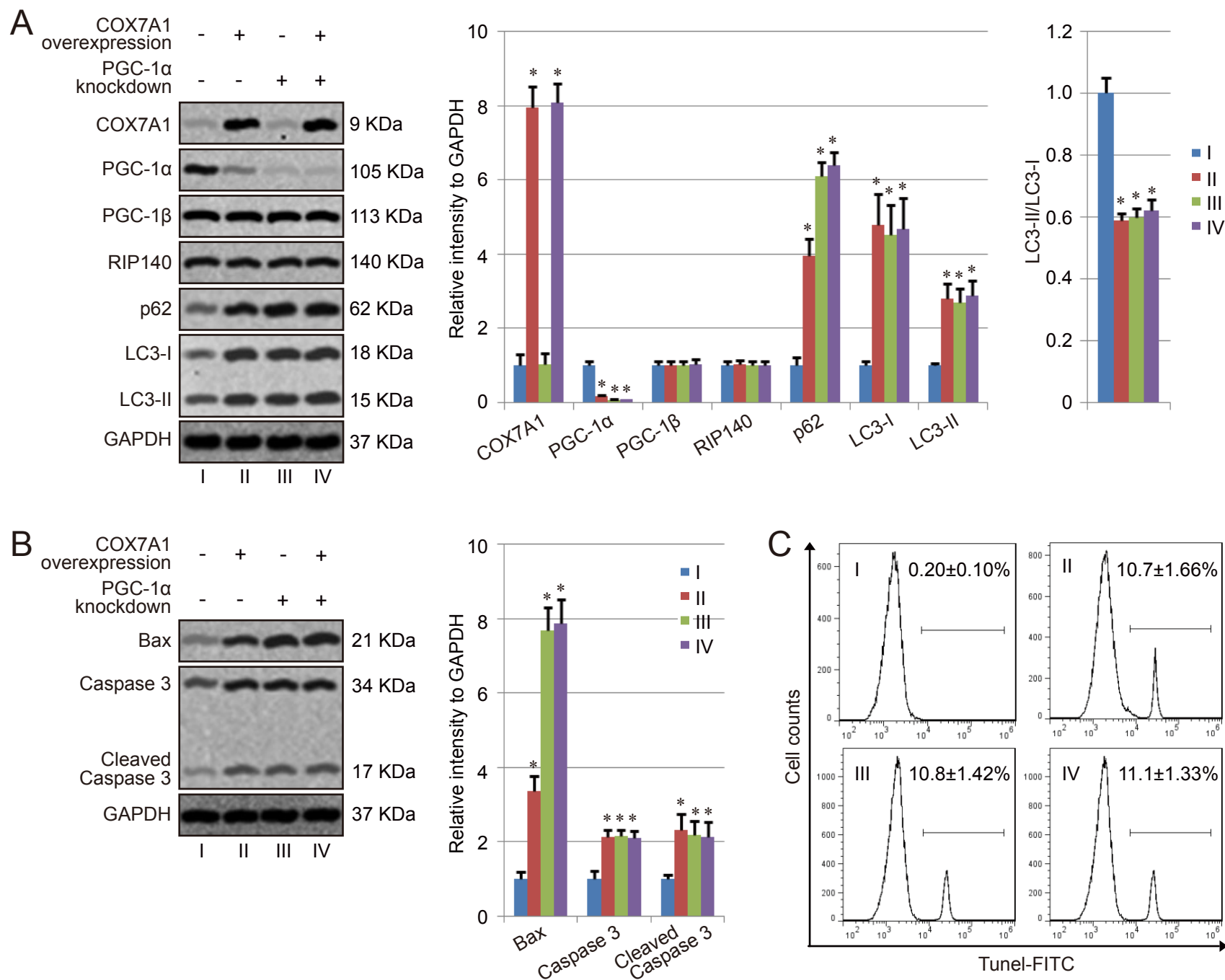

Supplement: Supplementary file 3 [file CAM4-8-7762-s003.pdf]

Figure S4

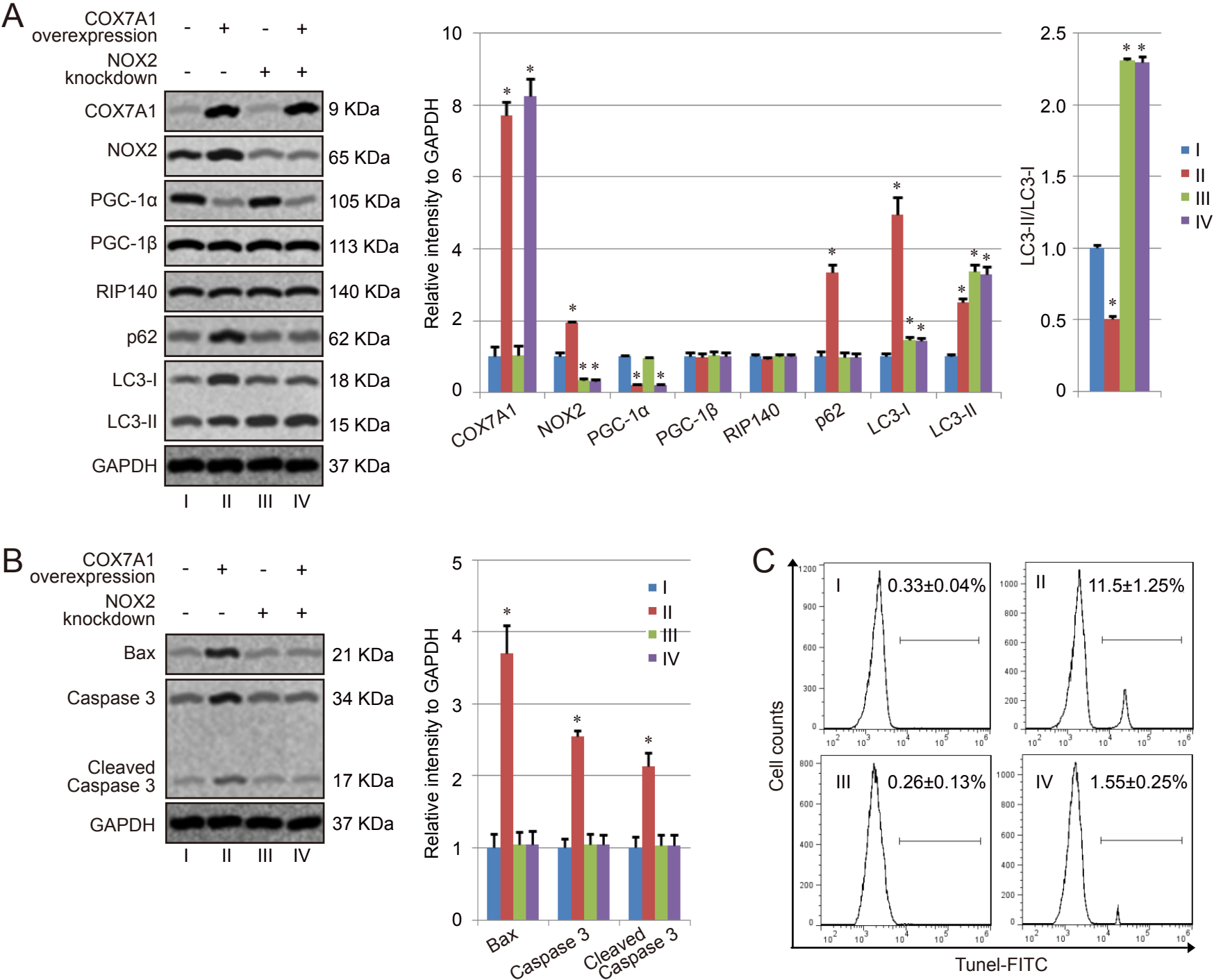

Supplement: Supplementary file 4 [file CAM4-8-7762-s004.pdf]
